# Empowering patients, empowering clinicians: How the lessons of HIV can inform chronic disease management across the primary healthcare system

**DOI:** 10.4102/sajhivmed.v18i1.692

**Published:** 2017-05-31

**Authors:** Andrea S. Mendelsohn

**Affiliations:** 1 The Desmond Tutu HIV Centre, University of Cape Town, South Africa

## Introduction

A 39-year-old virally suppressed man on antiretrovirals presented to Hannan Crusaid ARV Centre in Gugulethu with a complaint of frequent urination and weight loss. After telling him that he is a type 2 diabetic, he looked at me with concern and asked, ‘Can I pass this on to my daughter?’

Since moving to South Africa, I have been struck by how much healthcare and people’s understanding of health in South Africa is shaped by HIV. Clearly, the experience of so many young people dying was culturally traumatic for both ordinary citizens and healthcare providers alike. However, I have also been impressed by the public health infrastructure that has proactively and thoughtfully risen to respond to the epidemic. The crisis, and consequent national and international funding, gave South Africans a chance to stop and think of what the best way is to deliver chronic primary care services, rather than simply trying to get through the deluge of patients on a day-to-day basis. As a result, over three million people are receiving counselling, antiretrovirals (ARVs), adhering to medication and living positive healthy lives, not an insignificant accomplishment in a resource-limited context.

I think that many of the successes of HIV-related healthcare service delivery can be applied to the management of chronic diseases in South Africa more broadly by empowering both primary care clinicians and patients.

Empowering patients: That newly-diagnosed diabetic was concerned that – like HIV can pass from mother to child or tuberculosis (TB) within household – he could go home and infect his 3-year-old child with diabetes. His understanding of diabetes was poor, and his general health literacy level low, but his understanding of his HIV was excellent. He could recount how HIV is transmitted, what medications he took and the importance of adherence. He understood the concept of taking medication to lower blood sugar within the framework of taking ARVs to suppress one’s viral load. The three treatment readiness classes, HIV counselling and home visits had an impact – he understood the significance of the medication and was able to make his own healthcare decisions related to HIV from an informed vantage. I think that similar group education and treatment support, such as a culturally appropriate diabetes self-management curriculum taught by community health workers, could equally improve outcomes of other chronic diseases, including diabetes, asthma and hypertension. The days of paternalistically prescribing medication with minimal explanation are over. Patients need to be treatment ready and empowered for the self-management of all diseases. HIV has provided a model of how teams of healthcare workers, in collaboration with busy clinicians who often do not have the time for extensive education and community follow-up, can be locally viable and successful.

Simplifying treatment: One lesson from HIV is that less is more, decreasing pill burden is key to success. The same lesson could be applied to other primary care protocols. For instance, the current Western Cape guidelines favour starting a low dose diuretic and adding an angiotensin-converting-enzyme (ACE) inhibitor and then calcium channel blocker, before titrating any individual medication to the maximum dose. While scientifically this approach is valid, many patients end up on a higher pill burden than possibly necessary. Maximally titrating diuretics or calcium channel blockers as first-line agents might serve to decrease the overall pill burden of anti-hypertensives, thereby increasing adherence. Likewise, exploring generic combination anti-hypertensive agents in the public sector, such as combinations of diuretics and ACE-inhibitors, might also simplify patients’ regimens.

Empowering clinicians: By developing clear HIV/TB treatment guidelines and Nurse Initiated Management of Antiretroviral Therapy (NIMART) training programmes, nurses are able to provide the bulk of HIV/TB care in South Africa. This enables medical officers (MOs) to focus on complicated cases. MOs are given a fair amount of latitude to switch from first- to second- to even third-line regimens, with the approval of a specialist’s review only in the final case. In the case of non-communicable diseases, MOs are able to perform caesarean sections and stabilise trauma patients, but they are only allowed to prescribe first-line agents for basic medical conditions. Second-line agents and the corresponding laboratory investigations require the approval of a specialist. Primary care MOs should, for example, be able to prescribe proton pump inhibitors for short term gastro-oesophageal reflux symptoms not requiring a gastroscope, high dose corticosteroids for severe eczema not responding to weaker topicals, tamsulosin for men with history and physical examination consistent with benign prostatic hypertrophy, lipid lowering agents other than simvastatin in the case of drug-interactions (such as Aluvia), or long acting beta-agonists (LABAs) for uncontrolled asthmatics already on inhaled corticosteroids. Given the overall shortage of specialists and financial travel barriers for patients, such restrictions create a significant barrier to care. Many patients are left sub-optimally controlled because they are not sick enough to warrant a specialist referral or their care is unnecessarily fragmented making for a higher likelihood of errors.

General medical patients would benefit if primary care nurse-doctor teams were as empowered as their HIV colleagues. Nurses are highly capable of following detailed clinical protocols, such as the Western Cape’s 2015 Practical Approach to Care Kit (PACK),^1^ to prescribe first-line treatment and refer to MOs only for patients failing treatment or with multiple co-morbidities. MOs should be entrusted to appropriately prescribe a broader variety of agents and order medical work-ups at the primary care level, in consultation with a family physician or on-call specialists if necessary. MOs would still refer cases in which a specialist consultation or procedure was warranted. However, empowering primary care nurse-doctor teams to optimally medically manage their patients with the full range of treatment options available in the public sector would, at the very least, improve patient care by offsetting the demand for specialist referrals and might even arguably result in cost savings by keeping people healthier at baseline. If primary care is the heart of the South African system, give community nurses and doctors the tools to maximise their effectiveness and take ownership of their patients’ care.

## Conclusion

I am sure that primary care providers in South Africa have had dreams of improved delivery systems for years, filled with community health workers and educators all working together in a team to complement the clinician’s care. It is not as if anyone wants to keep prescribing medication after medication to an uncontrolled diabetic without seeing any improvement, yet not having the time to really figure out why the patient is not getting better or to discuss lifestyle changes. HIV was so tragic, that it forced South African healthcare workers to take the time to figure out a better way to deliver chronic care, a process that was enabled by massive international funding. This is not to say that HIV management is perfect, or the delivery of care cannot be improved. However, as an outsider looking in, it seems that South Africans used creativity and hard work to develop novel, multidisciplinary approaches to improve the management of a single chronic disease on a massive scale. Now is the time to apply those locally grown lessons more broadly to the treatment of all chronic diseases in the public sector.

## Footnotes

1 Primary Care Guidelines for Adults 2015, Western Cape Edition.

